# Emotional Discomfort Scale: Instrument Development and Association With General Self-Efficacy and Data From an Urban Primary Care Setting

**DOI:** 10.7759/cureus.21495

**Published:** 2022-01-22

**Authors:** Emmanouil K Symvoulakis, Panagiotis Volkos, Adelais Markaki, Manolis Linardakis

**Affiliations:** 1 Clinic of Social and Family Medicine/Faculty of Medicine, University of Crete, Heraklion, GRC; 2 Fourth Local Health Team, Academic Unit of Heraklion, Heraklion, GRC; 3 School of Nursing, University of Alabama at Birmingham, Birmingham, USA; 4 Department of Social Medicine/Faculty of Medicine, University of Crete, Heraklion, GRC

**Keywords:** instrument, psychometrics, self-efficacy, psychological distress, discomfort

## Abstract

Introduction: Sense of discomfort, which is experienced in daily encounters, can develop into stress, coexist with stress, or interplay with self-efficacy. This study presents two objectives, namely, to develop and test a new instrument called the Emotional Discomfort (EmoD) Scale and to compare the EmoD with the General Self-Efficacy (GSE) Scale.

Methods: The study was conducted in an urban primary healthcare center in Greece over a three-week period in 2020. Out of 314 individuals invited to participate, 263 accepted and completed the questionnaire. The EmoD is a five-point Likert-type eight-item scale for assessing individual reaction and sense of discomfort in daily life situations.

Results: Cronbach’s α for the new scale reached 0.730 (acceptable reliability). Participants who used psychotropic drugs scored higher in the EmoD scale compared with nonusers. GSE scores showed reverse associations with EmoD scores. Multiple linear regression analysis indicated that an increase in self-efficacy, as measured using the GSE scale, was associated with a reduction in sense of discomfort, as measured by the EmoD scale.

Conclusions: The use of the EmoD scale can aid health or social care providers in detecting levels of emotional discomfort, a finding that is demonstrated to interplay with self-efficacy. Future studies employing the use of this new instrument could examine emotional discomfort in relation to stress coping and social isolation.

## Introduction

Psychological or emotional distress (ED) is a state of emotional suffering or anguish typically characterized by symptoms of depression and anxiety [1]. This suffering is associated with stressors and demands, which are difficult to cope with in daily life and can be experienced at any time [2]. As a common mental health problem in the community, it manifests through a wide range of symptoms, such as the following: (1) feeling overwhelmed, helpless, or hopeless; (2) feeling guilty without a clear cause; (3) worrying; (4) having difficulty thinking or remembering; (5) excessive or lack of sleep; (6) changes in appetite; (7) heavy reliance on mood-altering substances; (8) isolation from people or activities; (9) unusual anger or irritability; (10) fatigue; (11) difficulty keeping up with daily tasks; and (12) experiencing new, unexplained pain [1].

ED is typically caused by a combination of factors. A traumatic experience or event, such as a death in the family, or particular circumstances at home or work, such as difficulties in relationships or financial strain, can trigger distress. It may also result from or may develop into a mental health disorder. The possible causes of ED at home include relationship problems with partners/family members/friends, major life changes (i.e., relocation or having a child), low income or debt, discrimination, living in a deprived or inequitable neighborhood, loneliness or isolation, and unhealthy lifestyle (i.e., smoking or lack of exercise) [3]. Work-triggered ED stems from concerns about job security or performance, long hours, low pay, poor working conditions, lack of control over work, increased responsibility, and relationships with colleagues or managers [3].

ED can become overwhelming and affect daily functioning. However, few studies have described the actual experiences of patients living with this condition. A phenomenological study from a primary care setting in Sweden demonstrated that persons with ED reported an imbalance between the self and the ideal self, which eventually diminishes self-esteem [2]. This imbalance was described as “struggling to cope with everyday life, feeling inferior, and losing one’s grip on life.” Given the difficulty in diagnosing ED and its potential for mental, physical, and emotional exhaustion, initiating preventive or early intervention becomes paramount for health professionals and patients. Although several tools for measuring the likelihood of anxiety and stress occurrence are available, such as the Perceived Stress Scale, State-Trait Anxiety Inventory, and the Beck Anxiety Inventory [4-6], a scarcity of tools focused on ED exists. During routine primary care visits, asking patients about recent experiences and major life events as potential triggers of ED will enable practitioners to implement techniques for stress reduction and alternative means of coping. Hence, using appropriate tools and training to systematically screen for ED and identify its triggers is critical.

The role of self-efficacy in addressing anxiety and psychological distress, especially in the literature on health psychology, has gained increased scholarly attention. Self-efficacy is the belief in one’s ability to succeed in a particular situation, which determines how people think, behave, and feel [7]. Although self-efficacy is not considered a personality trait, it is a situation-specific construct that determines which goals to pursue, how to accomplish these goals, and how to reflect on one’s performance. Previous studies have used self-efficacy theory to examine various behaviors, such as self-management of chronic diseases, smoking cessation, alcohol use, eating, exercise, and pain control. High levels of self-efficacy have been linked with resilience to adversity and stress, healthy lifestyle habits, improved employee performance, and educational achievement [8]. General self-efficacy is measured by the General Self-Efficacy (GSE) Scale, which was developed by Jerusalem and Schwarzer [9,10] to examine coping with stress and self-esteem, and was later linked to quality of life and health outcomes. As demonstrated, self-efficacy can influence perceived stress, health-related quality of life, and work-related stress [11-13]. Moreover, self-efficacy seemingly influences the manner in which people cope with or manage medical conditions, such as diabetes and cancer [14-16]. For adolescents with chronic conditions, self-efficacy was linked not only to physical but also to emotional and social quality of life [17]. For the elderly, Fry and Debats [18] suggest that self-efficacy could predict loneliness and psychological distress. In addition, loneliness was linked to the individual perception of stress, which suggests that people experiencing loneliness display high levels of stress [19]. Lastly, self-efficacy works as a protective factor against reusing alcohol and drugs, while higher levels of stress increase that reuse [20]. Since there is evidence in regards to specific substance use related to stress or self-efficacy, it would be interesting to examine the use of psychotropic drugs in conditions of routine uneasiness.

Against this background, the current study hypothesizes that individuals who experience a sense of emotional discomfort in daily encounters could rely on self-efficacy to mitigate and address such discomfort as normal uneasiness or expected unpleasantness. Evidence exists that several personality traits, such as extraversion, conscientiousness, and openness, are reversely associated with anxiety, depression, or substance disorders [21]. In addition, social anxiety is associated with personality traits, where high levels of openness serve as a protective factor when low levels of trust are considered [22]. However, less is known about the state of emotional discomfort in terms of whether it is a precursor to developing stress, coexists with stress, or interplays with self-efficacy. Hence, an instrument for measuring emotional discomfort on the basis of individual reaction to daily life events may provide a score for comparison with the GSE score. Therefore, the primary objective of this study was to develop and test the Emotional Discomfort (EmoD) scale and compare it with the GSE scale.

## Materials and methods

Setting and sample

The study was conducted at the Fourth Local Health Team (Topiki Omada Ygeias (TOMY)) of Heraklion, Crete, which is one of the 119 urban primary healthcare units composing the national health system of the country. The study population comprised all adult beneficiaries registered under TOMY (estimated at approximately 6,000). Beneficiaries with an appointment for any medical reason were eligible for the survey, provided that they (a) were over 18 years old, (b) did not seek urgent care, and (c) were able to cognitively and practically communicate the requested information. The sample was based on daily scheduled appointments over a span of three weeks in October 2020. Initially, 314 beneficiaries were invited to participate. However, only 263 agreed. Initially, the EmoD scale was completed by 16 individuals apart from the participants for test-retest reliability.

Ethics approval

The study was approved by the Ethics and Deontology Committee of the University of Crete (Protocol no. 176/25.09.2020) with consideration of the 1964 Helsinki Declaration and its later amendments and the 7th Health Regional Authority, Crete (Protocol no. 48814). The participants provided written informed consent.

Instruments

The EmoD scale was developed to capture emotional discomfort during common activities. The instrument comprised eight items for detecting how a person interacts with other people and reacts to routine communication stimuli (Table 1). The items were rated using a five-point Likert-type scale (1 = not at all to 5 = very much). For the pilot study, the reliability of the questionnaire was tested and retested using 16 participants who scored and rescored the tool within a two-week period. These participants were excluded from the final sample (n = 263). The mean score of the eight items of the two waves of completion provided a correlation coefficient of Pearson’s r = 0.80 (p < 0.001). Afterward, limited missing values for the self-completion of the scale were noted during data collection. Question 1 was the only exception. Missing data were detected from 17 participants who did not answer question 1. The study calculated the average scores for the eight items. High scores implied high levels of emotional discomfort, where values >3.00 are categorized under “more discomfort” (3.00 = moderate discomfort). Table 1 presents the final version after two sessions of back-translation.

**Table 1:** Emotional Discomfort (EmoD) Scale: after consensus, English version

1. Do you feel uncomfortable right before accessing your e-mail?
2. Do you feel uncomfortable when you miss a phone call with an unknown caller identification?
3. Do you feel uncomfortable when you eat at a public place?
4. Do you feel uncomfortable if you need to speak to a public audience?
5. Do you feel uneasy when someone scolds you?
6. Do you immediately feel the need to apologize to someone you have offended?
7. Do you feel uneasy if someone skips you in line in the bank or supermarket queue without asking?
8. Do you blush when you are uncomfortable?

Self-efficacy was measured using the Greek version of the GSE scale, which has been translated, culturally adapted, and validated [23]. The original scale consists of 10 items, which were designed to assess optimistic self-beliefs for coping with various life difficulties, and are rated using a four-point Likert-type scale [9,10]. No missing values were noted, whereas the total scores were calculated by summarizing the responses with composite scores ranging from 10 to 40 [24]. High scores indicate better self-efficacy and include participants with a score of 30.0 or more (the 67th percentile or above for the present sample).

Sociodemographic data (e.g., age, gender, health insurance, family status, employment, welfare access, and living expenses) were collected via a questionnaire, which also included items for smoking habits, sleeping routine, and the use and prescription of psychotropic drugs. According to the WHO, “psychotropic drugs” (currently called psychoactive) are those that influence mental processes, such as cognition or emotion [25]. The use of psychotropic drugs was extracted by inviting the participants to recall their prescriptions from the previous years. The participants could select from hypnotics, anxiolytics, antidepressants, antipsychotics, sedatives, none of the above, or “other” and provide the name of the prescribed medication. A prescription for one or more psychotropic drugs was regarded as “use.”

Statistical Analysis

Data were analyzed with the use of the SPSS software (IBM SPSS Statistics for Windows, Version 26.0 Armonk, NY: IBM Corp). The distributions of the descriptive characteristics of the participants were estimated, and comparisons were assessed with 95% confidence interval estimations. The reliability of the EmoD and GSE scales was estimated using the Cronbach method. Frequencies regarding the use of psychotropic drugs under the categories of “more discomfort” and “better self-efficacy” were compared using the Chi-square test (χ^2^). Based on hierarchical modeling, multiple linear regression analysis was performed for the EmoD scale in relation to the GSE scale and the characteristics of the participants. Significance was set to 0.05.

## Results

The majority or the 66.5% were females, while the mean age of all was 46.3 years (±14.5) or 43.4% with aged 50+; almost all of the participants (97.3%) were Greeks as well as individuals who are married or in a relationship reached 62.4%; and those with one or more children comprised 67.3%. Approximately half of the participants (52.1%) were employed, whereas the majority had health insurance (82.9%) (Table 2).

**Table 2:** Demographics of the participants (n = 263)

Characteristics		n	%
Gender			
Male		88	33.5
Female		175	66.5
Age in years mean (± SD) {min, max}	46.3	(±14.5)	{18, 78}
Age groups, in years			
<30		43	16.3
30–49		106	40.3
50+		114	43.4
Ethnicity			
Greek		256	97.3
Health insurance			
Yes		218	82.9
Occupation			
Employed		137	52.1
Unemployed, retired		126	47.9
Family status			
Married, in relationship		164	62.4
Unmarried, divorced, widow		99	37.6
Parity			
No child		86	32.7
1+		177	67.3

Moreover, significantly less than half or 35.0% were found as smokers (95% CI: 29.4, 40.9) with a mean consumption of 14.4 number of cigarettes per day. Approximately half of the participants (47.5%; 95% CI: 41.5, 53.6) reported interrupted sleep with a mean of 6.7 hours nocturnal sleep. A total of 19.0% reported a previous mental health diagnosis, whereas significantly less than a half or 26.6% (95% CI: 21.6, 32.2) declared the use of psychotropic drugs based on prescriptions from the previous year (Table 3).

**Table 3:** Health profile and habits of the participants (n = 263)

	n	%	95% CIs
Smoking habit			
No	171	65.0	59.1, 70.6
Yes	92	35.0	29.4, 40.9
Cigarettes/day mean (median) {min, max}	14.4	(13.5)	{1, 80}
Sleeping habit in hours/night mean (median) {min, max}	6.7	(7.0)	{3, 11}
Regular sleep through the night			
No	125	47.5	41.5, 53.6
Yes	138	52.5	46.4, 58.5
Mental health disorder diagnosis			
No	213	81.0	75.9, 85.4
Yes	50	19.0	14.6, 24.1
Use of prescribed psychotropic drugs			
None	193	73.4	67.8, 78.4
At least one	70	26.6	21.6, 32.2

The EmoD and GSE scales had acceptable reliability (α > 0.7) (Table 4). According also to the categorization of EmoD (based on 3.00+ limit), significantly less than a half of participants or 31.6% (95% CI: 26.2, 37.4) experienced “more discomfort”, whereas about a half in GSE (based on 30+ limit) reported “better self-efficacy” (49.0%; 95% CI: 43.0, 55.1).

**Table 4:** Emotional Discomfort (EmoD) versus General Self-Efficacy (GSE) score measurements (n = 263) ^a^EmoD scale range is based on eight questions with responses categorized as 1 = not at all; 2 = little, 3 = moderate, 4 = much, and 5 = very much. High scores imply high levels of discomfort, whereas values >3.00 are characterized as “more discomfort” (3.00 is considered moderate). ^b^GSE scale range is 10–40 based on 10 questions with responses categorized as 1 = not at all true to 4 = exactly true. High scores indicate high levels of self-efficacy, whereas a score of 30.0 or more indicates better self-efficacy (the 67th percentile) where score of 30.0 was found in n = 27 participants (10.3%).

Scale	Mean	Standard deviation	Median	Cronbach α
EmoD Scale^a^	2.64	0.70	2.63	0.730
More discomfort, 3.00+ n (%) {95% CI}	83	(31.6)	{26.2, 37.4}	
GSE Scale^b^	29.0	5.23	29.0	0.868
Better self-efficacy, 30+ n (%) {95% CI}	129	(49.0)	{43.0, 55.1}	

**Figure 1:**
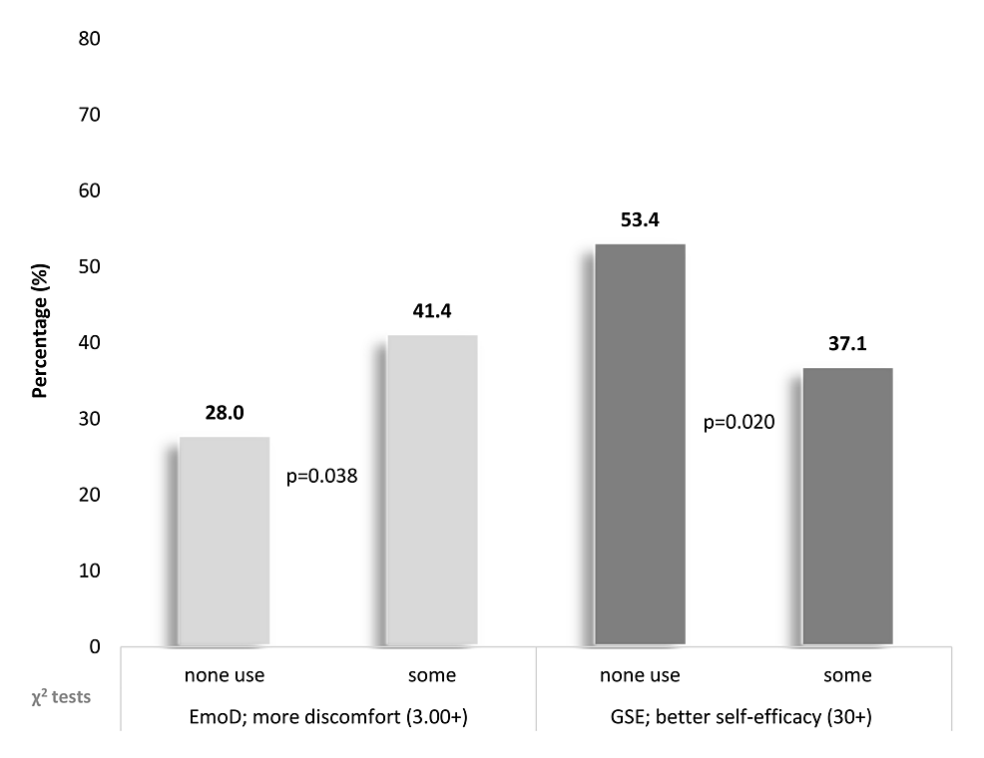
Distribution (%) of the use of psychotropic drugs under “more discomfort” (EmoD scale) and “better self-efficacy” (GSE Scale) (n = 263) EMoD Scale: Emotional Discomfort Scale, GSE Scale: General Self-Efficacy Scale.

However, the distribution of the aforementioned groups according to the use of psychotropic drugs indicated a reverse relationship between the two scales (Figure 1). Specifically, the proportion of participants under the categories “use” of psychotropic drugs and “more discomfort” was significantly higher compared with those with “no use” of psychotropic drugs (41.4% vs. 28.0%, p = 0.038). On the contrary, the number of participants grouped under the categories “use” of psychotropic drugs and “better self-efficacy” was significantly lower compared with those with “no use” of psychotropic drugs (37.1% vs. 53.4%, p = 0.020).

Finally, in a multivariate level, Table 5 presents the hierarchical modeling of multiple linear regression analysis on the EmoD scale in relation to the GSE scale and the characteristics of the sample as prognostic factors. In the first model, mental health diagnosis and the use of psychotropic drugs and/or characteristics were analyzed, and there was not found any significant relationship between EmoD scale and these factors (p > 0.05). The second model analyzed the EmoD and GSE scores and found a significant association. Specifically, an increase in self-efficacy was significantly associated with a reduction in emotional discomfort (β = −0.03, p = 0.001).

**Table 5:** Hierarchical modeling with multiple linear regression analysis of EmoD scale in relation to GSE scale and participant characteristics (n=263) β or betas: unstandardized regression coefficients, EMoD Scale: Emotional Discomfort Scale, GSE Scale: General Self-Efficacy Scale.

	Emotional Discomfort (EmoD) Scale			
	First model		Second model	
Variables	β	p-value	β	p-value
Gender (1: male, 2: female)	0.08	0.370	0.03	0.736
Age (years)	0.00	0.753	0.00	0.519
Ethnicity (1: Greek, 2: other)	0.04	0.893	0.07	0.802
Insurance (1: no, 2: yes)	−0.11	0.402	−0.11	0.379
Occupation (1: employed, 2: unemployed, retired)	0.14	0.141	0.10	0.289
Family status (1: married, in relationship, 2: unmarried, divorced, widow)	−0.03	0.747	−0.02	0.805
Parity (number of children)	−0.01	0.902	−0.01	0.884
Smoking habit (1: no, 2: yes)	−0.08	0.368	−0.09	0.322
Regular sleep through the night (1: no, 2: yes)	−0.03	0.707	−0.04	0.665
Mental health disorder diagnosis in the past (1: no, 2: yes)	0.17	0.243	0.07	0.624
Use of psychotropic drugs (1: none, 2: at least one)	0.13	0.307	0.14	0.282
General Self-Efficacy Scale (10–40)	–	–	−0.03	0.001
R^2^ (adjusted)	0.063	(0.022)	0.107	(0.064)

## Discussion

The main objective of the study was to preliminarily assess a new scale, namely, EmoD, which intends to determine the state of emotional discomfort during common daily encounters. The newly developed instrument, namely, EmoD, exhibited a Cronbach’s α value (0.730) within the acceptable range. In the multivariate analysis, GSE found to fairly contribute to the model explanation in comparison to other factors. Unstandardized β regression factor was estimated at a p < 0.001, and its size is of limited matter in terms of interpretation. Additionally, the study endeavored to explore whether the EmoD score and GSE score were related to the previous use of psychotropic drugs. From those with high emotional discomfort, participants who reported some use of psychotropic drugs were significantly more than those who reported no use (p = 0.038). From those with high self-efficacy, participants who reported no use of psychotropic drugs were more than those who reported some use (p = 0.020).

Questions one, two, and three detect feelings of uneasiness, ruled by elements of passive involvement during routine activities. Due to this discomfort, a person may be prepared to handle a situation with nonpredictable dimensions (questions one and two) or moments out of the ordinary privacy practices (question three). Questions four, five, six, seven, and eight detect feelings of uneasiness in circumstances ruled by elements of a more active interaction or involvement, in terms of direct exposure at a situation that can be complicated or is perceived as embarrassing. Due to this discomfort, a person may install a status of preparedness or alertness. Ingredients of uncertainty, uneasiness, awareness, or preparedness are present in all eight questions of the scale, with a different mixture but with a common perception of discomfort. This was the theoretical motivation to consider the summation of the scale.

Weidner et al. [26] reported a positive association between self-efficacy and self-management of menopause symptoms. Similarly, organ donation awareness was positively correlated with high GSE scores [24]. On the contrary, self-efficacy was negatively associated with professional burnout, as reported by Makara-Studzińska et al. [27]. Recently, Hong et al. [28], in a large prospective, nationally representative study on adults in the United States, aged >50 years demonstrated that a sense of control was mainly related to better health behaviors, higher psychological well-being, and lower psychological distress. Interestingly, the authors found that sense of control was unrelated to lifestyle habits (e.g., binge drinking and smoking) and social factors (e.g., living with spouse/partner and frequency of contact with children and other family members). The authors concluded that a sense of control should be targeted as a potential intervention for fostering physical, behavioral, and psychosocial health. Given that EmoD captures meanings of emotional discomfort, with a possible unknown linkage with sense of control, a plausible hypothesis emerging from the current study is that the new scale can offer some elements of routine uneasiness, awareness, or self-containment to be tested within a primary care or social care setting in order to provide early explanations of how persons deal with routine feelings that may influence their decisions.

The study of Tate et al. [20] indicated that people with high levels of self-efficacy and low levels of stress demonstrated increased resistance to the reuse of substances, such as alcohol, cannabis, or stimulants. Additionally, self-efficacy was positively associated with the intention to limit alcohol use, which may result in less consumption of alcohol in the future [29]. According to a household survey on Crete in 2012, every family had at least one medication stored at home, where 17% of the drugs are antibiotics [30]. Therefore, linking emotional discomfort with other psychosocial variables is a thought which may add views to explore ruling motivations in regards to drug or substance consumption.

Patients with ED are characterized by the inability to cope, changes in emotional status, discomfort, and communication of discomfort and harm [31]. Drawing from social cognitive theory, self-efficacy can be established through mastery experiences, social modeling, social persuasion, and psychological responses [32]. Therefore, patients need to become aware of and discuss with primary healthcare professionals how to strengthen their capacities to regain health and well-being [2].

Amid increasing evidence that individuals with high levels of spirituality are less likely to develop depression, hypertension, diabetes mellitus, and cardiovascular conditions [33,34], future studies could explore the possible correlations between emotional discomfort and spirituality. Moreover, such studies could examine the possible associations between the scores for the EmoD scale and the Personal Sociability and Connections Scale, which measures the development of social connections in daily life [35].

Limitations and strengths

Due to the cross-sectional design, the study cannot establish causality. Self-reporting bias cannot be excluded as eventuality since the questionnaire for the sociodemographic and health characteristics of the participants was self-administered with no access to the electronic health records of the patients. Lastly, the study established the association between EmoD and the use of psychotropic drugs. Further research is required to explore the use and the associations of the EmoD scale with factors that interplay with decision making related to health. Further initiatives will also help to gain responses on how emotional discomfort and psychotropic drugs mutually interact overtime.

## Conclusions

Emotional discomfort, which may be experienced in daily activities, can develop into stress, coexist with stress, or interplay with self-efficacy. The newly designed instrument can be used in primary care or social care settings and research initiatives. The scale allows healthcare providers to capture levels of emotional discomfort of individuals in an attempt to understand how they cope with daily uneasiness or well-being, given the reversed correlation between GSE and EmoD. Lastly, EmoD can be used in everyday practice to explore elements of psychotropic drug use. Future studies that employ this new instrument could examine emotional discomfort in relation to stress coping and social interaction.
